# Heterogeneous Colistin-Resistance Phenotypes Coexisting in *Stenotrophomonas maltophilia* Isolates Influence Colistin Susceptibility Testing

**DOI:** 10.3389/fmicb.2018.02871

**Published:** 2018-11-22

**Authors:** Sònia Martínez-Servat, Daniel Yero, Pol Huedo, Roser Marquez, Gara Molina, Xavier Daura, Isidre Gibert

**Affiliations:** ^1^Institut de Biotecnologia i de Biomedicina, Universitat Autònoma de Barcelona, Barcelona, Spain; ^2^Departament de Genètica i de Microbiologia, Universitat Autònoma de Barcelona, Barcelona, Spain; ^3^Catalan Institution for Research and Advanced Studies, Barcelona, Spain

**Keywords:** colistin, susceptibility testing, heteroresistance, adaptive resistance, biofilm

## Abstract

The polymyxin antibiotic colistin shows *in vitro* activity against *Stenotrophomonas maltophilia*. However, an increased incidence of colistin-resistant isolates has been recently observed. In addition, *in vitro* evaluation of colistin susceptibility for this organism has been problematic. The aims of this study were to investigate the colistin-resistance phenotypes displayed by *S. maltophilia* and their potential association with the challenging determination of colistin susceptibilities for this organism by even the recommended method. Colistin-resistance phenotypes were inferred by use of the recommended broth microdilution method in different clinical isolates of *S. maltophilia*. Most of the strains showed non-interpretable minimum inhibitory concentrations (MICs) for colistin due to an incomplete growth inhibition in wells of the microdilution plate. In addition, the subpopulation of bacteria resistant to colistin showed an increased ability to form biofilms on the plastic surface of MIC plates. The observed incomplete growth inhibition in the microdilution plates is compatible with a progressive adaptation to colistin or a heterogeneous susceptibility to this antibiotic. Therefore, to determine the existence of heteroresistance or adaptive resistance, four colistin-resistant clinical isolates were subjected to serial Etest assays, growth rate analyses, and the population analysis profile test. The experiments indicated that these *S. maltophilia* isolates display a colistin-resistant sub-population that survives and multiplies in the presence of the antibiotic. Interestingly, this phenomenon might not be explainable by the natural background mutation rate alone since the development of a resistant sub-population occurred upon the contact with the antibiotic and it was reversible. This complex colistin-resistance phenotype is exhibited differently by the different isolates and significantly affected colistin susceptibility testing. Furthermore, it can coexist with adaptive resistance to colistin as response to pre-incubation with sub-inhibitory concentrations of the antibiotic. Overall, the combined action of heterogeneous colistin-resistance mechanisms in *S. maltophilia* isolates, including colistin-induced biofilm formation, may hamper the correct interpretation of colistin susceptibility tests, thus having potentially serious implications on antimicrobial-therapy decision making.

## Introduction

*Stenotrophomonas maltophilia* has recently been recognized as a significant opportunistic pathogen in a number of healthcare settings (Gherardi et al., 2015). An alarming trait of this species is that most isolates display intrinsic or acquired resistance mechanisms to a large number of antibiotic classes. Furthermore, a trend toward increased resistance to antimicrobials and higher frequencies of multidrug resistant (MDR) isolates have been observed during the last years (Rutter et al., 2017). Although monotherapy with trimethoprim-sulfamethoxazole or tetracyclines such as tigecycline, minocycline, and doxycycline remains the most effective antimicrobial treatment against this organism (Chang et al., 2015), combination therapies and novel agents are currently needed to treat infections caused by MDR strains. Colistin has proven to be active against *S. maltophilia* *in vitro* (Gales et al., 2006; Rodríguez et al., 2014; Poirel et al., 2017) and effective for the treatment of patients in combination with other drugs (Wood et al., 2010). However, an increased incidence of colistin-resistant isolates has also been observed in recent years (Juhász et al., 2017).

In Gram-negative bacteria, colistin resistance may arise through several mechanisms (Olaitan et al., 2014), such as regulated modifications of the LPS molecule, mutations in genes involved in LPS synthesis or variations in global gene expression induced by environmental changes such as variations in pH or cations or the presence of cationic antimicrobial polypeptides (Groisman et al., 1997; Fernández et al., 2010). In addition, different phenotypic resistance mechanisms acting cooperatively in bacterial populations, such as adaptive resistance (Fernández et al., 2010), heteroresistance (Falagas et al., 2010), and biofilm formation (Kim et al., 2015; Chua et al., 2016), have accelerated the emergence of colistin resistance. Heterogeneous resistance to colistin, a phenomenon by which different sub-populations within a single isolate exhibit various susceptibilities to this antibiotic, is a growing clinical problem in association to MDR Gram-negative pathogens (Barin et al., 2013; Band et al., 2016; Halaby et al., 2016; Hjort et al., 2016). Although there are controversies and some overlaps in the definition of concepts, adaptive resistance and heteroresistance have been distinguished by the temporality and reversibility of each phenomenon. While both phenotypes lead to sub-populations with heterogeneous susceptibilities to a particular antimicrobial agent, heteroresistance has been referred to as the coexistence of genetically different sub-populations within a single isolate, with phenotypes that are heritable by several generations (Band et al., 2016). On the other hand, adaptive resistance has a transient nature and involves a short-lived increase in the ability of a bacterium to resist an antibiotic treatment, due to alterations in gene expression as a result of exposure to sub-inhibitory levels of the antibiotic itself or other stressors (Fernández and Hancock, 2012).

One important virulence-related trait of some bacteria is their ability to form biofilms, which among other functions facilitates bacterial persistence and resistance to the action of antimicrobials (Stewart, 2015), including colistin (Mulcahy et al., 2008; Kim et al., 2015; Chua et al., 2016), or to the immune system. This is the reason why biofilm formation has also been considered as a form of phenotypic resistance (Olivares et al., 2013). *S. maltophilia* is known to form biofilms on a wide range of biotic and abiotic surfaces including indwelling medical devices (De Vidipó et al., 2001; de Oliveira-Garcia et al., 2003; Pompilio et al., 2008). There are several methods for biofilm antimicrobial susceptibility testing (Macia et al., 2014) that have contributed to the determination of effective antibiotic treatments against these bacteria under biofilm-formed conditions (Di Bonaventura et al., 2004; Wu et al., 2013; Wang et al., 2016).

The precise mechanisms of polymyxin resistance in *S. maltophilia* have been poorly studied and, currently, there is a dearth of data regarding heteroresistance or adaptive resistance to colistin in this microorganism. In addition, the determination of clinical minimum inhibitory concentration (MIC) breakpoints for colistin has been typically inconsistent for *S. maltophilia*, not only due to the inaccuracy and unreliability of susceptibility testing methods recommended for this antibiotic and bacteria (Nicodemo et al., 2004; Gülmez et al., 2010; Moskowitz et al., 2010; Betts et al., 2014), but also because of the high genetic diversity of the clinical isolates of this species (Gherardi et al., 2015). The aforementioned facts motivated the study presented here on the resistance phenotypes of colistin-resistant isolates of *S. maltophilia*. Our work reveals a complex resistance behavior involving the concerted action of different colistin-resistance mechanisms, leading to the failure of colistin-susceptibility testing methods on *S. maltophilia*.

## Materials and Methods

### Bacterial Isolates

The primary *S. maltophilia* strain used in this study was K279a. It is a reference clinical MDR strain shown to be resistant to colistin (Youenou et al., 2015). In addition, a panel of 58 clinical and reference isolates of *S. maltophilia* was included in the study. The name of the additional strains can be found in Supplementary Figure S1. The clinical isolates were collected between 1998 and 2012 from point prevalence studies in ICUs of geographically distant European hospitals (Huedo et al., 2014). This panel included isolates from sputum, blood, and swabs from surgical wound, oropharynx, perineum, vascular ulcer, decubitus ulcer, or a bronchoaspirate. The model strain D457 was also included and it was kindly provided by Dr José L. Martínez (National Centre for Biotechnology, Madrid, Spain). Strain ATCC 13637 was included as references, and it was purchased from the American Type Culture Collection.

### Colistin Susceptibility Testing

Minimum inhibitory concentration of colistin was determined by the broth microdilution (BMD) method in accordance with CLSI/EUCAST recommendations (Clinical and Laboratory Standards Institute, 2015; The European Committee on Antimicrobial Susceptibility Testing and Clinical and Laboratory Standards Institute, 2016). Briefly, MICs were determined in sterile 96-well plates by twofold serial dilutions of colistin sulfate (Apollo Scientific Ltd., cat. No. BIC0118, Batch AS405305) in cation adjusted Mueller Hinton broth (CAMHB). To prepare CAMHB, Mueller Hinton broth (MHB) from Oxoid (cat. No. CM0405. Thermo Fisher Scientific, Basingstoke, United Kingdom) was supplemented with calcium and magnesium to final cation concentrations of 25 and 12.5 mg/L, respectively. An antibiotic stock solution was diluted in CAMHB to 2× the top concentration used in the test (512 mg/L), and it was used to prepare plates containing 100 μl of twofold serial dilutions in each well. Bacteria were first grown overnight in CAMHB using CLSI-recommended incubation conditions. After that, 100 μL of bacterial suspensions with a final optical density at 550 nm (OD_550_) of 0.005 were added to the wells containing the 2× antibiotic dilutions, and the MIC plates were read after 20 h of incubation at 37°C. To determine MIC end points, several approaches were taken into account. The lowest concentration showing no bacterial growth as evaluated by visual inspection was initially taken as the MIC value, and cell viability was confirmed by addition of 30 μl of 0.01% resazurin to each well (Sarker et al., 2007). Any color alteration from blue to purple or pink was recorded as positive and, in this case, the MIC was defined as the lowest drug concentration which prevented this color change. In addition to visual MIC evaluation and before adding resazurin, the OD_550_ and the number of colony forming units (CFU) were determined in each well. The CFU per mL were estimated by plating serial dilutions of the bacterial suspension. If it was not possible to accurately discern growth in the wells by visual inspection and resazurin staining, the MIC was defined as the lowest antibiotic concentration that inhibited 80% of growth (based on OD measurements) in comparison to the positive growth control without antibiotic (Clinical and Laboratory Standards Institute, 2015). These calculations were based on four independent replicates. The susceptibility test results were interpreted according to susceptibility and resistance clinical breakpoints suggested by the EUCAST (European Committee on Antimicrobial Susceptibility Testing, 2018) for *Pseudomonas sp.* (susceptible, MIC ≤2 mg/L; resistant, MIC >2 mg/L).

MIC for colistin was also determined using Etest strips following the manufacturer’s instructions (bioMérieux, Madrid, Spain). A 0.5 McFarland suspension from an overnight culture in CAMHB was used to create a confluent bacterial lawn on in-house prepared Mueller-Hinton agar (MHA) plates using agar powder from Oxoid (Thermo Fisher Scientific, Basingstoke, United Kingdom). The MIC values were determined according to the Etest reading guide after 20 h of incubation at 37°C. The MIC was read where inhibition of growth intersected the Etest strip. When small colonies grew within the zone of inhibition, the highest MIC intersect was recorded.

### Biofilm Formation in Broth Microdilution Susceptibility Plates

Quantification of the biofilm biomass in the MIC microdilution plate was performed by crystal violet (CV) staining as described previously (Huedo et al., 2014). Briefly, wells were washed three times, fixed at 60°C for 1 h and stained for 15 min with 200 μL of CV solution at 0.1%. The stained biofilms were rinsed with distilled water, allowed to dry at 37°C for 30 min and then extracted with 200 μL of 30% acetic acid. The amount of biofilm was quantified by measuring the OD_550_ of dissolved CV using a microplate reader (Multilabel Plater Reader VICTOR3). Biofilm formation was normalized by cell growth and reported as relative biofilm formation. Furthermore, a colorimetric resazurin-based protocol was used for the detection of biofilm viability (Toté et al., 2008). Briefly, growth medium was discarded from established biofilms in MIC plates, and remaining adherent cells were washed three times with PBS. Wells were filled with 200 μL of MHB and 50 μL of resazurin (0.5 μg per well). After incubation at 37°C for 50 min, fluorescence intensity was measured using a VICTOR3 spectrophotometer at excitation/emission wavelengths of 531/572 nm. For background correction, wells containing only culture medium and treated with resazurin were used. For these quantitative assays, we have used four replicate wells for each treatment. Statistical values (*P* values) were calculated using the one-way analysis of variance (ANOVA) test and Dunnett’s post-test software packages in GraphPad Prism 5 (GraphPad Software, Inc.).

### Adaptive Resistance to Colistin

For the analysis of adaptive resistance to colistin, the growth curves of selected isolates were determined after preincubation with colistin. Overnight cultures (10 mL) in CAMHB were grown with agitation (200 rpm) at 37°C with or without sub-MIC colistin concentrations (concentration depending on the strain). The next day, fresh cultures in CAMHB were prepared from the overnight cultures (initial OD_550_ = 0.01), and colistin at a concentration equal to or above the calculated MIC values for each strain was added to these suspensions. Flasks were continuously shaken (200 rpm) at 37°C. Fresh cultures without colistin were prepared as controls. To determine the bacterial growth with and without the antibiotic, aliquots were taken at specified intervals to determine the OD_550_. All experiments were carried in duplicate in at least two independent tests.

### Heteroresistance to Colistin

The heteroresistance to colistin was investigated in selected strains by the population analysis profile (PAP) assay as described previously (Li et al., 2006) with some modifications. Briefly, MHA testing plates were prepared by adding 0, 2, 4, 8, 16, 32, 64, 128, 256, or 512 mg/L of colistin. Ten to fifty microliter aliquots of eight separate bacterial inoculums from tenfold serial dilutions from an overnight culture (adjusted to ∼10^9^ CFU/ml) were spread onto the testing plates. Three or four replicate experiments were performed for each strain. Colonies were counted on each plate after 24 h of incubation at 37°C, and the criteria suggested by El-Halfawy and Valvano (2015) were applied to determine heteroresistance. An isolate was considered as heteroresistant when the lowest antibiotic concentration giving maximum growth inhibition was >eightfold higher than the highest non-inhibitory concentration determined by BMD. Furthermore, colonies that grew on PAP plates containing the highest concentrations of colistin were picked and sub-cultured daily for 3 days in antibiotic-free CAMHB. MICs for colistin in these sub-populations were determined by the BMD method as described above.

## Results

### Challenges Associated to Colistin Susceptibility Testing in *S. maltophilia* by the Recommended BMD Method

**FIGURE 1:**
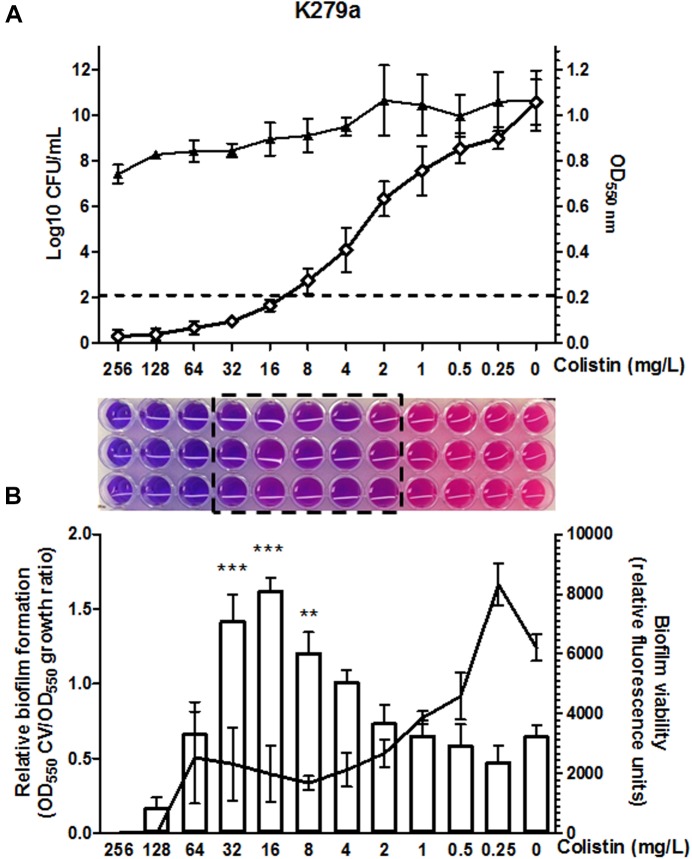
Colistin MIC determination by the broth microdilution method in CAMHB for the clinical reference strain K279a. **(A)** The optical density at 550 nm (white diamond) and CFU/ml (black triangle) in each well of the microtiter plate was measured after incubation at 37°C. Values are means of four replicates and error bars indicate the standard deviation. Under the graph, the photograph of triplicate MIC determinations in a 96-well microdilution plate using the colorimetric indicator resazurin shows the purple/pink transition zone (boxed) between colistin concentrations of 32 and 2 mg/L. Colistin concentrations are in serial twofold dilutions starting at 256 mg/L. The colistin MIC, taken as the lowest concentration that inhibited 80% of growth as compared to the control well without antibiotic (dashed line in the graph), was 16 mg/L. **(B)** Effect of colistin concentration on the attachment of the cells to the surface of the microtiter wells and on cell survival in the formed biofilm. Total biofilm formation relative to bacterial growth in each well is shown as a bar graph (*Y* axis on the left) and the viability of the biofilm determined by resazurin staining as a line graph (*Y* axis on the right). Fluorescence units are expressed as relative to the biofilm formed in each well of the microtiter plate. For each experiment values are means of four replicates and error bars indicate the standard deviation. The significant differences between biofilm formation values were calculated using one-way ANOVA with Dunnett’s multiple comparison test. Statistically significant increases with respect to untreated control are indicated: ^∗∗^*P* < 0.01 and ^∗∗∗^*P* < 0.001.

Colistin susceptibility was first investigated in *S. maltophilia* reference strain K279a using the BMD method following CLSI recommendations. This strain was primarily chosen based on discrepancies between the results obtained by different colistin susceptibility testing methods at the time of reception at our laboratory. Although BMD is considered the reference method for polymyxin susceptibility testing, the accurate interpretation of colistin MIC values in the microdilution plates was challenging for this strain. The susceptibility breakpoint was inferred initially by direct visual examination of bacterial growth, resulting in non-interpretable MIC determinations for this isolate. Colistin plates were then evaluated with the resazurin method. Once again, determination of MIC breakpoints for colistin was difficult, due to the appearance of a “transition zone” in wells displaying incomplete growth inhibition, resulting in an ambiguous purple/pink color when stained with resazurin (Figure 1A). Bacterial burden in wells of the MIC plate confirmed incomplete bacterial growth inhibition in the purple/pink transition zone between colistin concentrations of 32 and 2 mg/L (Figure 1A), suggesting the existence of mechanisms at the population level generating heterogeneous colistin resistance. For this strain, viable bacteria could be obtained at antibiotic concentrations even higher than those in the resazurin-stained transition zone (Figure 1A). The same challenge associated to colistin MIC determination by the BMD method for strain K279a was observed in other *S. maltophilia* strains. In a panel of 61 strains (including K279a), the purple/pink transition zone in the MIC plate was present in 78.7% of the isolates including both colistin-sensitive and -resistant strains (Supplementary Figure S1). From this set of strains, an incomplete growth in the transition zone, assessed by counting the number of CFU in each well, was also confirmed in the three resistant *S. maltophilia* isolates M30, D457, and PG157 (Table 1 and Supplementary Figure S2). In addition to K279a, these three resistant isolates were selected fur further studies.

**Table 1:** Colistin susceptibilities (mg/L) of the four selected resistant *S. maltophilia* strains determined by the BMD and Etest methods, and interpretation issues related to both methods.

Strain	BMD method		Etest method	
	MIC^a^	Transition zone in BMD^b^ (concentration range)	MIC^c^	Isolated colonies inside the inhibition halo (concentration range)
K279a	16	Yes (2–32)	4	Yes (0.125–4)
M30	32	Yes (8–128)	48	Yes (0.125–48)
D457	32	Yes (16–>256)	128	Yes (1.5–128)
PG157	32	Yes (8–64)	0.5	Yes (0.125–0.5)


*^a^MIC was defined as the lowest antibiotic concentration that inhibited 80% of growth (based on OD measurements) in the BMD plate. ^*b*^Wells in the BMD plate displaying incomplete growth inhibition and thus resulting in an ambiguous purple/pink color when stained with resazurin. ^*c*^When small colonies grew within the zone of inhibition, the highest MIC intersect was recorded.*

Finally, and in order to define a breakpoint, the MIC value was inferred by measuring the optical density at 550 nm of each well as proposed by the CLSI guidelines. For strain K279a the colistin MIC thus measured was found 16 mg/L, corresponding to the lowest concentration value in the microdilution plate exhibiting ≥80% growth inhibition compared to the absorbance of the well without antibiotic (Figure 1A). We then used this criterion to determine colistin MIC values for all the isolates tested here by the BMD method (Table 1 and Supplementary Figure S1). By this method, the colistin resistance rate in our set of 61 isolates was 67.3% with a MIC50 value above the resistant breakpoint (>2 μg/mL).

Since *S. maltophilia* is recognized by its ability to form biofilms on a plastic surface in microtiter plates, we next evaluated the adherence of the tested isolates to the wall of the wells in the colistin MIC plates. Interestingly, colistin at concentrations around the MIC value significantly increased the ability of the resistant sub-populations of strains K279a, M30, D457, and PG157 to form live biofilms (Figure 1B and Supplementary Figure S2). Specifically, the strain K279a exhibited the highest relative biofilm formation at antibiotic concentrations in the purple/pink transition zone. As determined with resazurin, colistin at concentration of 64 mg/L completely killed the cells of this strain in the biofilm after 24 h of incubation. Higher colistin concentrations effectively prevented biofilm formation *in vitro* by the resistant sub-population.

### Colistin Susceptibility Testing in *S. maltophilia* by the Etest Method Is Hampered by Heterogeneous Resistance to Colistin

**FIGURE 2:**
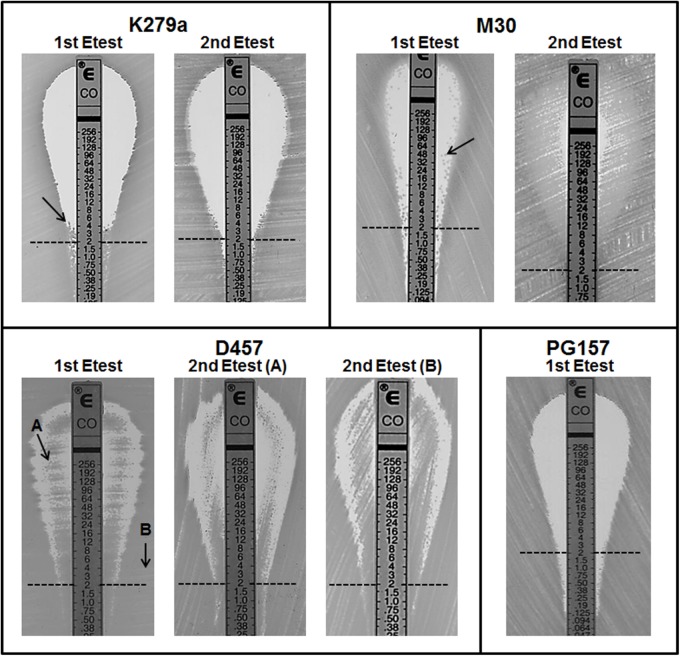
Result of colistin Etest for four isolates of *S. maltophilia* showing resistance to colistin by the broth microdilution method. Colistin concentrations are indicated in mg/L. For each isolate, an isolated colony within the zone of inhibition (indicated with an arrow) was taken for a second colistin Etest. For strain D457 cells were taken from either the inner (sample A) or outer (sample B) growth zone for the second Etest. In the first Etest, PG157 displayed sharper end point reading without visible resistant colonies. The clinical breakpoint for resistance (2 ml/L) is indicated with a dashed line.

Although colistin MIC testing by other methods including gradient diffusion is not recommended, colistin susceptibility was also investigated by the Etest method for the four colistin resistant strains K279a, M30, D457, and PG157. As expected (Moskowitz et al., 2010), the results of the BMD and Etest methods were significantly different for all the strains (Table 1). For instance, for the strain PG157 the Etest gave a colistin MIC value of 0.5 mg/L that corresponds to susceptible strains (breakpoint ≤ 2 mg/L), whereas the BMD method yielded a colistin MIC value (32 mg/L) above the resistant breakpoint. In addition, the Etest plates seeded with the different colistin-resistant isolates showed different reading patterns, mainly characterized by the emergence of isolated colonies within the inhibition ellipse (Figure 2). Curiously, after incubation of the Etest plates, strain D457 showed a double halo of growth within the inhibition zone. For strains K279a and M30, the growth of discrete resistant colonies at different antibiotic concentrations was observed. In particular, small resistant colonies of strain K279a grew within the zone of inhibition at concentrations lower than 4 mg/L, while additional colonies appeared at higher colistin concentrations only after more than 24 h of incubation. Strain M30 showed resistant colonies within the inhibition zone at higher antibiotic concentrations (up to 96 mg/L). Conversely, the strain PG157 displayed only discrete colonies in the bottom of the ellipse and below the susceptibility breakpoint (2 mg/L), despite significant differences between the Etest and BMD results (Figure 2). In general, the growth of resistant colonies within the inhibition zone in the Etest assays has been proposed as an indicator of heteroresistance (El-Halfawy and Valvano, 2015).

To assess whether the detected heteroresistance phenotype was reversible, isolated resistant colonies taken from the inhibition zone of colistin Etest plates of isolates K279a, M30, or D457 were re-grown to perform a second Etest assay (Figure 2). These colonies were immediately streaked onto fresh MH agar plates. For strain K279a, when one of the colonies that grew close to the 4 mg/L concentration point was streaked onto a new fresh colistin Etest plate, resistant colonies did not appear within 24 h of incubation (Figure 2). This transient nature of the resistance phenotype suggested the presence of an adaptive resistance mechanism rather than heteroresistance. For strain M30, a resistant colony close to the 48 mg/L concentration point was picked for the second Etest. As expected for a heteroresistant strain, its colistin-resistance capacity was maintained in the second assay, with a MIC value for the whole population even higher than the original resistant colony (Figure 2). Finally, a single colony from the atypical inner ring of growth observed for strain D457 was picked for a second Etest, in which the double halo was surprisingly maintained (Figure 2), suggesting that the colistin-resistant sub-population was not a permanent phenotype. For this strain, we repeated the Etest several times and obtained always the double halo for colonies isolated from either the inner or outer growth zones (data not shown).

### Collective Phenomena Involved in Colistin Resistance in *S. maltophilia*

To further investigate bacterial growth and possible adaptive resistance, the four strains were pre-incubated with a sub-MIC colistin concentration. Strains D457 and M30 showed evidence of adaptation after pre-incubation with 1.0 mg/L colistin, but strains K279a and PG157 required fourfold more colistin to adapt. After pre-incubation, cells were challenged with the minimum antibiotic concentration that inhibited the growth of the non-adapted cells. The growth curves (Supplementary Figure S3) suggested the development of adaptive resistance in all strains tested, although colistin at inhibitory concentrations reduced the growth of the pre-induced cultures for all strains except D457. These results indicate that pre-incubation of the bacterial cells with sub-inhibitory colistin concentrations may either induce the resistance of a sub-population to higher antibiotic concentrations or select for a pre-existing resistant subpopulation. Strikingly, strain D457 was able to grow, independently of the pre-induction conditions, at colistin concentrations below 256 mg/L (data not shown), and a sub-population of this strain started to resist the effect of colistin at 256 mg/L after 6 h of incubation even in the absence of pre-inducing conditions. Importantly, bacterial growth was observed for all isolates in the non-induced overnight cultures treated with an inhibitory concentration of colistin, after 24 h incubation. This result points to a pre-existing resistant subpopulation or to the adaptation of a small fraction of the population upon the first contact with the antibiotic.

**FIGURE 3:**
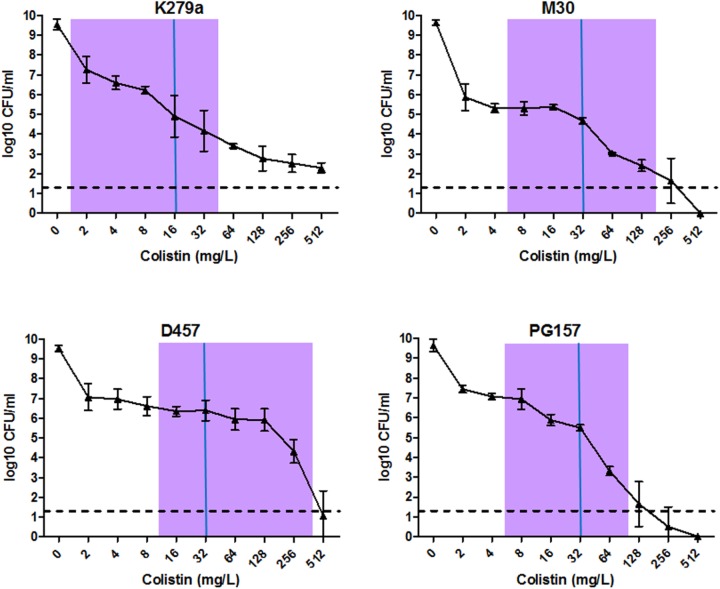
Analysis of hetero-resistant subpopulations of selected *S. maltophilia* isolates by population analysis profile (PAP). PAP was determined at 24 h by agar dilution of cultures exposed to serial dilutions of colistin. The *y* axis indicates the number of colonies on MHA plates, and concentrations of colistin are shown on the *x* axis. Values are means of three or four replicate experiments with SD. The limit of counting was 20 CFU/ml, and it is indicated with a horizontal dashed line. For each strain, MIC value calculated by the BMD method is indicated with a solid vertical blue line and a purple bar indicates the resazurin-stained transition zone.

Finally, to corroborate the existence of colistin-resistant sub-populations in the strains K279a, M30, PG157, and D457, the PAP analysis was performed (Figure 3). Although these strains showed MIC values of 16–32 mg/L in the BMD assay (Table 1), the PAP analysis revealed the existence of resistant sub-populations growing at antibiotic concentrations eightfold higher than their highest non-inhibitory concentrations. These results strongly suggested the presence of heteroresistance in all strains. To investigate whether this resistance phenotype was reversible, several colistin-resistant clones were picked from the PAP plates with the highest antibiotic concentrations, sub-cultured into liquid antibiotic-free medium, and subjected to new MIC determination by BMD. Interestingly, for all strains tested, most of the apparently heteroresistant clones restored their susceptibility to colistin to the original levels. These results indicate that the heterogeneous resistance phenotype may be reversible in these *S. maltophilia* strains and is not only the result of heritable stable mutations, as implied by the traditional heteroresistance concept. The finding that heteroresistance in *S. maltophilia* may also be of transient nature, certainly adds more complexity to the multifactorial resistance phenotype. Nonetheless, truly heteroresistant subpopulations, meaning stable resistant clones, were also generated in the strains K279a, M30, and PG157. These stable resistant clones were picked from PAP plates with colistin concentrations higher than the upper limit of the resazurin-stained transition zone for each isolate (Figure 3). The proportion of stable resistant clones was on the order of 10^-7^ to 10^-8^, which is within the natural mutation-rate range reported for this species (Turrientes et al., 2010).

## Discussion

As for other Gram-negative bacteria (Grote et al., 2015), phenotypic heterogeneity has also been observed in clinical *S. maltophilia* isolates, with effects on resistance to antibiotics, such as β-lactams or quinolones (Abda et al., 2015; Pak et al., 2015), among other phenotypes. In the present study, we have shown for the first time that *S. maltophilia* is also capable of developing colistin-resistance heterogeneity, as a result of the joined action of different phenotypes of resistance to this antibiotic at the population level. Our results initially suggest that both population-based strategies, heteroresistance and adaptive resistance, can coexist in *S. maltophilia*, as observed for the resistant strains K279a, M30, PG157, and D457. However, the impact and predominance of each of these mechanisms in global resistance to colistin may vary greatly among isolates. Even in the genetically very close isolates M30 and D457 (Huedo et al., 2014), the heterogeneous resistance phenotype turned out to be markedly different. In strain D457 in particular, the heterogeneous response to colistin is strongly evident, especially with the Etest method. Conversely, for some other strains tested (Supplementary Figure S1), including both colistin-sensitive and -resistant isolates, there was no indication of heteroresistance or adaptation to colistin in the BMD assays, suggesting that some *S. maltophilia* isolates may not have such collectively operated resistance mechanism to colistin.

Heteroresistance was identified in resistant isolates by Etest and subsequent PAP analysis and was not related in these experiments to previous exposure to colistin, although we have shown in parallel that these strains have the capacity to adapt to this antibiotic. However, for the strains tested, the concept of heteroresistance may not be strictly applied because a resistant *S. maltophilia* sub-population lost colistin resistance in antibiotic-free medium. A similar phenotype has also been described in clinical isolates of *Pseudomonas aeruginosa* (Lee et al., 2016) and *Enterobacter cloacae* (Band et al., 2016). For the latter bacterium, authors referred to this resistance phenomenon as to “clonal heteroresistance,” and they attributed it to a minor antibiotic-resistant sub-population that is capable of replicating in the presence of colistin and mediating resistance to high levels of the antibiotic. This resistant sub-population was not the result of a stable mutation, and it was selected very early during the first exposure to the antibiotic (Band et al., 2016), as for example in the susceptibility tests. This is probably what is happening in the *S. maltophilia* isolates displaying heterogeneous resistance to colistin in this study. Our results also suggest that an initial resistant sub-population emerges as a consequence of transient phenotypic changes. The question of whether this combined phenotypic manifestation has occurred within a genetically homogeneous strain is something that requires further investigation, including genomic sequencing and transcriptome-based analyses of consecutive isolates under antibiotic stress. Transposon mutagenesis and transcriptome analysis have revealed the mechanisms governing adaptive resistance in *P. aeruginosa* and *Acinetobacter baumannii* (Fernández et al., 2010, 2012; Hood et al., 2013). Two-component regulators and genes involved in lipopolysaccharide synthesis and modification were identified as the drivers of inducible colistin resistance. On the other side, amino acid substitutions in the two-component systems PmrAB, PhoPQ, or ParRS have been associated with the emergence of colistin heteroresistance in these species (Choi and Ko, 2014).

The incapacity to correctly determine MIC values for colistin when dealing with *S. maltophilia* is surely a consequence of the aforementioned population-based resistance mechanisms. Inconsistent data for the *in vitro* activity of colistin against *S. maltophilia* isolates have also been reported in other studies and likely stem from discordance between different testing methods (Nicodemo et al., 2004; Gülmez et al., 2010; Moskowitz et al., 2010; Betts et al., 2014). In addition, although BMD currently remains the reference assay for determination of MICs (Clinical and Laboratory Standards Institute, 2015; Poirel et al., 2017), the occurrence of a “transition zone” with this method when determining susceptibility to colistin must be taken as a warning. Non-interpretable MICs for some isolates are evidently due to the growth of resistant sub-populations in wells of the microdilution plate containing low antibiotic concentrations. MIC determination by visual inspection or by measuring the optical density of each well in the BMD method should be reconsidered and determination of the minimum bactericidal concentration should be taken into consideration for *S. maltophilia*.

To make the determination of colistin MICs for *S. maltophilia* even more complex, sub-inhibitory colistin concentrations are found to increase the adherence of the surviving resistant sub-population to plastic surfaces. The biofilm formed in the wells of the MIC plates could not only affect the accuracy of MIC-value determination but also the occurrence of the “transition zone” in the BMD method. Previous studies have demonstrated that *S. maltophilia* biofilm formation greatly reduces sensitivity to certain antibiotics (Liaw et al., 2010). On the other hand, it has already been reported that a sub-lethal dose of some antibiotics can increase biofilm formation by pathogenic organisms (Hoffman et al., 2005; Nguyen et al., 2014; Knudsen et al., 2016). However, to the best of our knowledge, here we show for the first time that colistin can increase biofilm formation *in vitro* in *S. maltophilia*, mainly in the colistin-resistant sub-population after a challenge with the antibiotic.

## Conclusion

Overall, this study shows that recommended susceptibility testing methods for colistin in *S. maltophilia* may lead to unreliable results, mainly due to a complex interaction between different resistance mechanisms in the bacterial cells, including both adaptive resistance and heteroresistance. This heterogeneous susceptibility to colistin is present in both resistant and sensitive isolates and could arise in part as a consequence of transient phenotypic changes activated quickly upon the contact with colistin. All this together could have important implications in clinical laboratories, particularly for antimicrobial-therapy decision making. According to the results shown here, colistin alone should not be considered a routine therapeutic option against *S. maltophilia* due to its capacity to rapidly adapt to this antibiotic, although it should not be ruled out as a last-resort drug in combination therapies. The potential clinical significance and therapeutic implications of heterogeneous resistance to colistin in *S. maltophilia* certainly calls for more experimental and clinical research on the topic.

## Author Contributions

SM-S, DY, PH, RM, and GM performed the experiments presented in the work. SM-S and DY did the main writing of the article. All authors contributed to the design and interpretation of the results, revised the article, and approved it for publication.

## Conflict of Interest Statement

The authors declare that the research was conducted in the absence of any commercial or financial relationships that could be construed as a potential conflict of interest.
