# Supplementary material for: Heterogeneous Colistin-Resistance Phenotypes Coexisting in Stenotrophomonas maltophilia Isolates Influence Colistin Susceptibility Testing
Source: Front Microbiol. 2018 Nov 22;9:2871. doi: 10.3389/fmicb.2018.02871 (PMC6262003; doi:10.3389/fmicb.2018.02871)
Supplement: Supplementary file 1 [file Data_Sheet_1.PDF]

Supplementary Material

Heterogeneous colistin-resistance phenotypes coexisting in *Stenotrophomonas maltophilia* isolates influence colistin susceptibility testing

Sònia Martínez-Servat, Daniel Yero, Pol Huedo, Roser Marquez, Gara Molina, Xavier Daura* and Isidre Gibert*

***Correspondence:** Corresponding Authors Xavier.Daura@uab.cat and Isidre.Gibert@uab.cat

Supplementary figures

	Colistin (mg/L)										
	Resistant							Susceptible			
Strains	256	128	64	32	16	8	4	2	1	0.5	0.25
Strains showing heteroresistant response to colistin											
PU140	←MIC										
OU141	←MIC										
PC187	MIC										
PR45		MIC									
EV5520			MIC								
PC257			MIC								
PC255			MIC								
P626				MIC							
M30				MIC							
PL20				MIC							
D457				MIC							
PG157				MIC							
B5565				MIC							
PR59				MIC							
PC232				MIC							
K279a					MIC						
E50					MIC						
PC314					MIC						
B285						MIC					
UV74						MIC					
PC312						MIC					
OL13						MIC					
PC274						MIC					
S5720						MIC					
OS87						MIC					
PU101						MIC					
OU148						MIC					
PC186						MIC					
OC194						MIC					
PC313						MIC					
PC240							MIC				
OS91							MIC				
P815							MIC				
ATCC13637							MIC				
PC245								MIC			
OR41								MIC			
PU126								MIC			
4834-R									MIC		
OC197									MIC		
OL16									MIC		
PU109									MIC		
OU353									MIC		
PC226									MIC		
OU110										MIC	
PU153										MIC	
OC323										MIC	
OU343										MIC	
H5726											MIC→
Strains showing homogeneous response to colistin											
S3149			MIC								
OR79				MIC							
OL11					MIC						
PU100					MIC						
S289						MIC					
B5742							MIC				
PU102							MIC				
OG156								MIC			
PL12										MIC	
OU111											MIC
OU152											MIC
E77											MIC→
L9-5R5											MIC→

Supplementary Figure S1. Graphical representation of the resazurin-based 96-well plate BMD method for the determination of the minimum inhibitory concentration (MIC) of colistin in a panel of 61 *S. maltophilia* strains. The color changes were observed 30 min after addition of resazurin (pink color indicates growth and blue means inhibition of growth). The purple color in the graph indicates the purple/pink "transition zone". The MIC was taken as the first well in the microdilution plate exhibiting 80% growth inhibition compared to the absorbance (OD550) of the well without antibiotic (arrows indicate MIC values out of the detection limits). The clinical breakpoint for resistance (2 ml/L) is indicated with a thicker black line.

D457

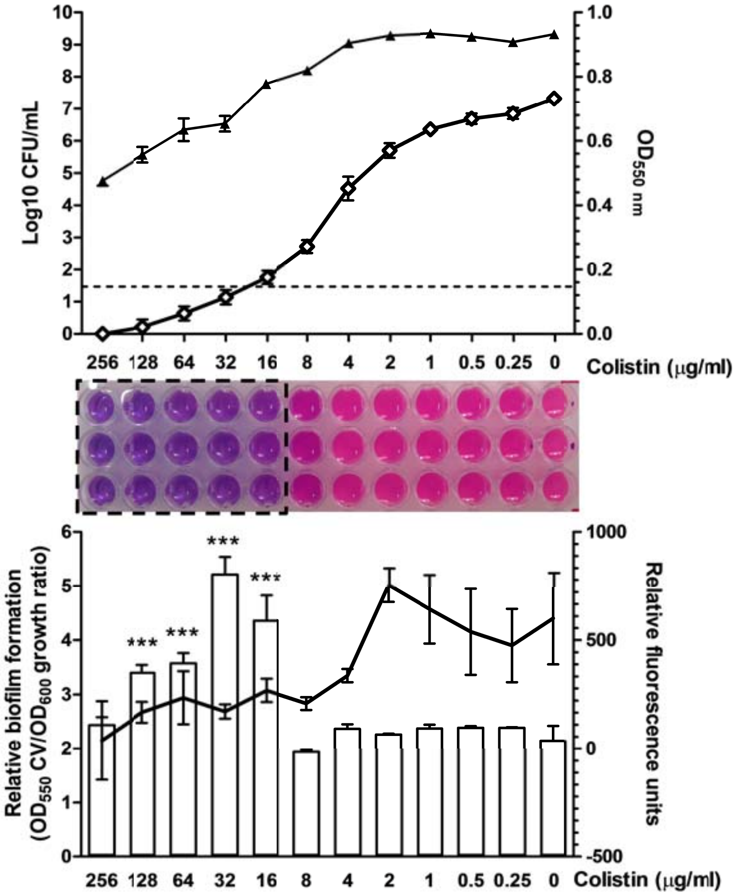

Log10 CFU/mL
OD$_{550\ nm}$
256 128 64 32 16 8 4 2 1 0.5 0.25 0
Colistin (μg/ml)
Relative biofilm formation (OD$_{550}$ CV/OD$_{600}$ growth ratio)
Relative fluorescence units
***
***
***
***

M30

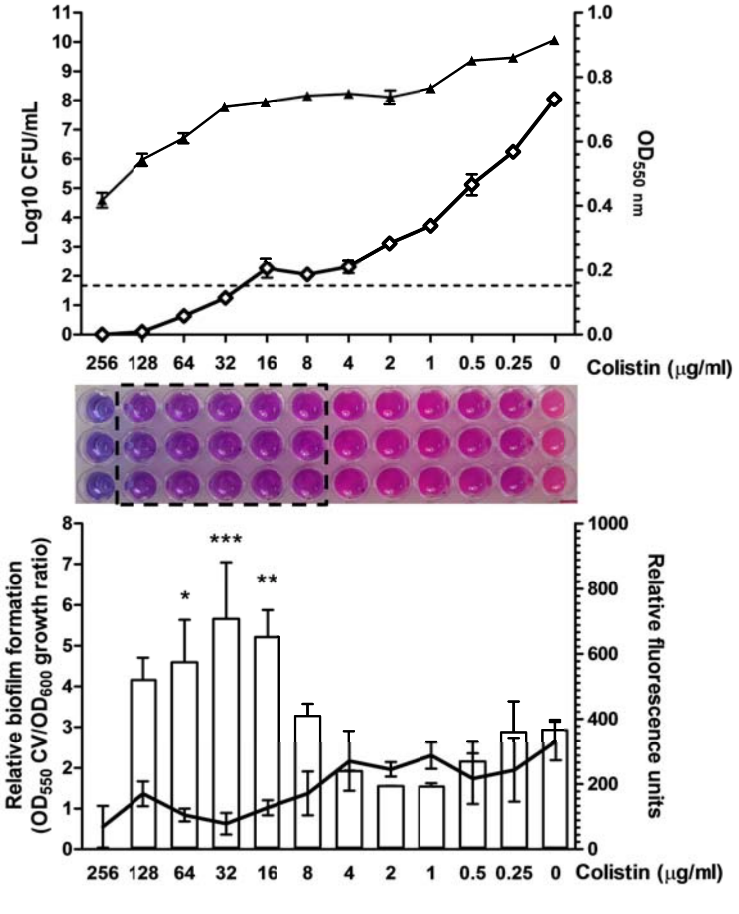

Log10 CFU/mL
OD$_{550\ nm}$
256 128 64 32 16 8 4 2 1 0.5 0.25 0
Colistin (μg/ml)
Relative biofilm formation (OD$_{550}$ CV/OD$_{600}$ growth ratio)
Relative fluorescence units
*
***
**

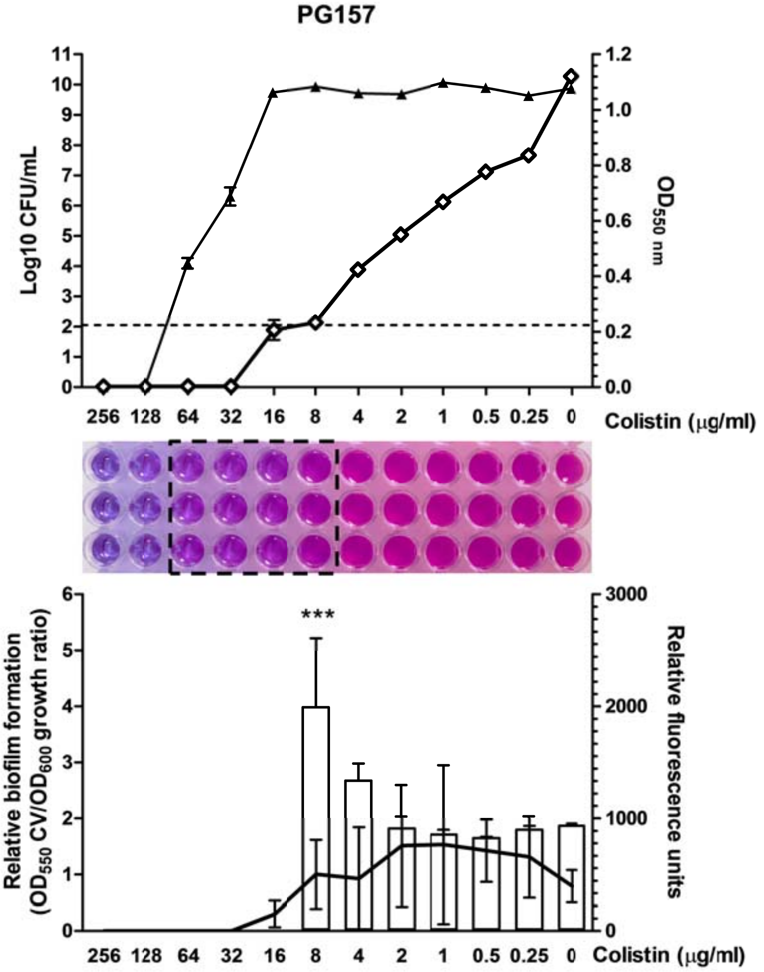

Supplementary Figure S2: Uninterpretable results during colistin MIC determination by the BMD method in CAMHB broth for the colistin-resistant clinical strains D457, M30 and PG157 (Upper graphic in each panel). The optical density at 550 nm (white diamond) and CFU/ml (black triangle) of each well in the microtiter plate was measured after 24 hours of incubation at 37°C. Error bars are generated from three replicates. Colistin concentrations are in serial two-fold dilutions starting at 256 mg/L. The colistin MIC was taken as the lowest concentration that inhibited 80% of growth (OD550) as compared to the control well without antibiotic (dashed line in the graph). A picture of three replicas of the microdilution wells with the calorimetric indicator resazurin is shown below each graph with the purple/pink transition zone boxed. The lower graphic in each panel shows total biofilm formation relative to bacterial growth in each well of the BMD plate as a bar graph (Y axis on the left) and the viability of the biofilm determined by resazurin staining as a line graph (Y axis on the right). Fluorescence units are expressed as relative to the biofilm formed in each well of the microtiter plate. Error bars are generated from four replicates in each experiment. Statistically significant increases in biofilm formation with respect to untreated control are indicated: * $P<0.05$, ** $P<0.01$ and *** $P<0.001$ (one-way ANOVA with Dunnett's multiple comparison test).

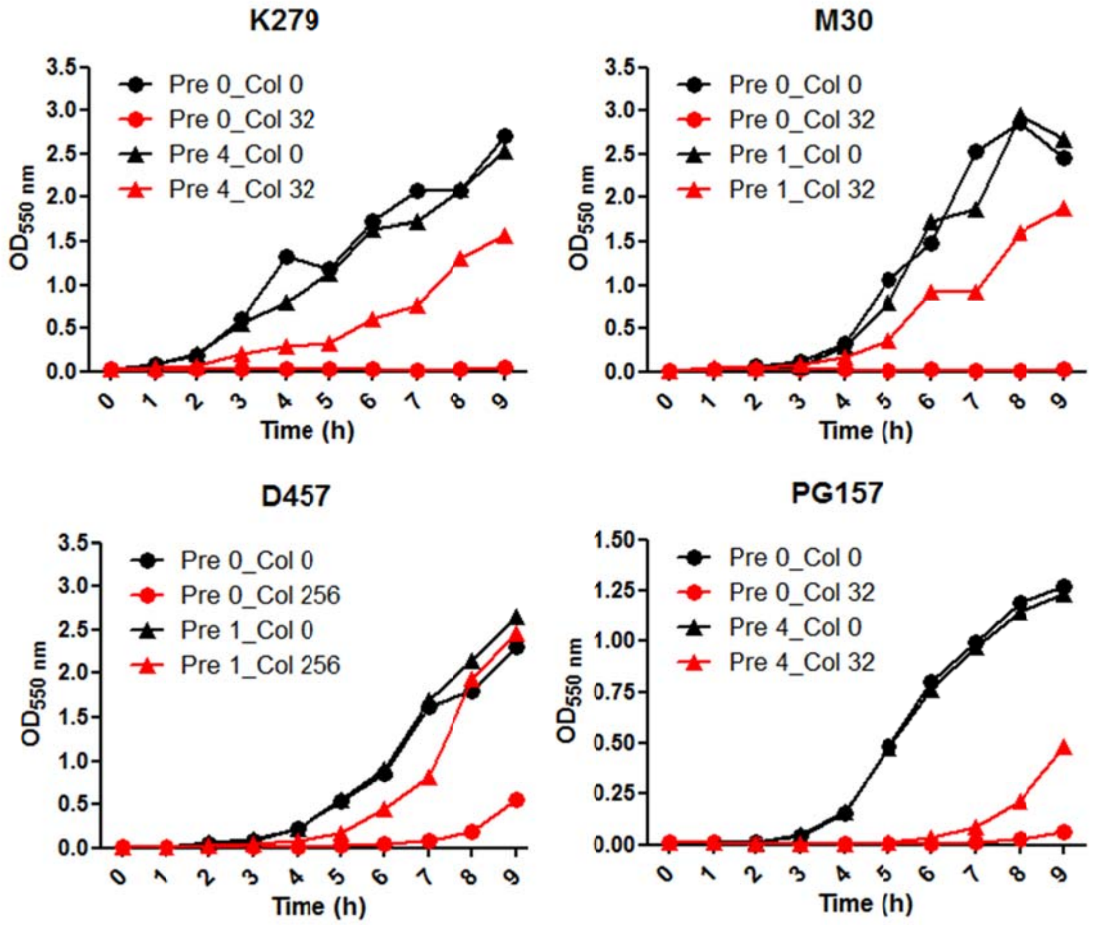

Supplementary Figure S3: Adaptive response to colistin in four isolates of *S. maltophilia* showing resistance to colistin by the broth microdilution method. Effect of preincubation with sub-inhibitory concentrations of colistin (Pre in mg/L) on the growth of selected strains in CAMHB supplemented with an inhibitory concentration of colistin (Col in mg/L).
